# Effects of Metformin Monotherapy Versus Metformin Plus SGLT-2 Inhibitor Therapy on Molecular and Cellular Inflammatory Markers in Patients with Newly Diagnosed Type 2 Diabetes Mellitus

**DOI:** 10.3390/jcm15176700

**Published:** 2026-08-29

**Authors:** Bennur Esen, Damla Yildiz, Ahmet Engin Atay

**Affiliations:** Bağcılar Training and Research Hospital, 34200 Bagcilar, Turkey

**Keywords:** type 2 diabetes mellitus, metformin, SGLT-2 inhibitor, inflammation, TNF-α, IL-6, systemic immune-inflammation index, systemic inflammation response index

## Abstract

**Background/Objectives:** Type 2 diabetes mellitus (T2DM) is a metabolic disease characterized by insulin resistance and chronic low-grade inflammation. Tumor necrosis factor-alpha (TNF-α), interleukin-6 (IL-6), the systemic immune-inflammation index (SII), and the systemic inflammation response index (SIRI) are important markers used to evaluate inflammatory status in T2DM. This study aimed to compare inflammatory and metabolic parameters between patients receiving metformin monotherapy and those receiving metformin plus sodium-glucose cotransporter-2 (SGLT-2) inhibitor combination therapy in patients with newly diagnosed T2DM. **Methods:** This prospective observational study included 64 treatment-naïve patients with newly diagnosed T2DM. Patients were divided into two groups according to treatment strategy: metformin monotherapy (*n* = 32) and metformin plus SGLT-2 inhibitor combination therapy (*n* = 32). TNF-α, IL-6, SII, systemic inflammation response index (SIRI), neutrophil to lymphocyte ratio (NLR), C-reactive protein (CRP), metabolic parameters, and insulin resistance-related indices were evaluated at baseline and after six months. Baseline-adjusted multiple linear regression analyses were performed to assess between-group differences at six months, adjusting for the corresponding baseline biomarker value, baseline HbA1c, age, and sex. **Results:** After six months of follow-up, significant reductions in TNF-α, IL-6, glycated hemoglobin (HbA1c), and fasting plasma glucose levels were observed in both groups (*p* < 0.001). In baseline-adjusted analyses, the treatment-group association was not statistically significant for TNF-α (B = −26.80, β = −0.15, *p* = 0.213), CRP (B = 0.06, β = 0.00, *p* = 0.977), SII (B = −36.94, β = −0.11, *p* = 0.441), or NLR (B = −0.20, β = −0.18, *p* = 0.207). In contrast, the treatment group was associated with a lower six-month IL-6 level (B = −37.87, β = −0.31, *p* = 0.038; 95% CI, −73.52 to −2.23) after adjustment for baseline IL-6, baseline HbA1c, age, and sex. Serum uric acid levels were lower in the metformin plus SGLT-2 inhibitor group than in the metformin monotherapy group at six months in the unadjusted comparison (*p* = 0.016). However, the adjusted treatment-group association between treatment group and six-month serum uric acid levels was not statistically significant (B = −0.51, β = −0.20, *p* = 0.065). **Conclusions:** In patients with newly diagnosed T2DM, both treatment groups showed significant reductions in TNF-α and IL-6 levels over six months. After adjustment for baseline biomarker levels, baseline HbA1c, age, and sex, no significant incremental association with treatment group was detected for TNF-α, CRP, SII, or NLR, whereas a significant adjusted association was observed for IL-6. Given the observational design and physician-directed treatment allocation, the observed IL-6 association should not be interpreted as evidence of a causal anti-inflammatory effect of SGLT-2 inhibitor therapy.

## 1. Introduction

Type 2 diabetes mellitus (T2DM) is a complex metabolic disease characterized by insulin resistance, β-cell dysfunction, and chronic low-grade inflammation. Tumor necrosis factor-alpha (TNF-α), one of the major proinflammatory cytokines involved in obesity-related metabolic inflammation, contributes to peripheral insulin resistance by impairing insulin signaling [1]. Furthermore, TNF-α has been shown to disrupt endothelial insulin signaling, thereby promoting vascular inflammation and endothelial dysfunction [2].

Interleukin-6 (IL-6) is an important proinflammatory cytokine associated with insulin resistance [3]. Experimental studies have shown that chronic IL-6 exposure may contribute to insulin resistance through inflammatory mechanisms [4]. TNF-α and IL-6 are considered key mediators involved in the development and maintenance of metabolic inflammation [5].

Metformin, in addition to its glucose-lowering properties, suppresses inflammatory signaling pathways, decreases the expression of proinflammatory cytokines such as TNF-α and IL-6, and improves insulin sensitivity [6,7].

Sodium-glucose cotransporter-2 (SGLT-2) inhibitors are increasingly used in the treatment of T2DM due to their ability to reduce renal glucose reabsorption, improve glycemic control, and provide cardiovascular and renal protective benefits [8,9]. Beyond their glucose-lowering properties, SGLT-2 inhibitors have been associated with changes in inflammatory pathways in experimental and clinical studies by reducing oxidative stress and improving mitochondrial function [10]. However, their associations with inflammation-related biomarkers have not been consistent across clinical studies. In a study evaluating empagliflozin, serum IL-6 levels were significantly reduced, whereas no significant changes were observed in TNF-α and interleukin-1 beta (IL-1β) levels [11].

The systemic immune-inflammation index (SII) and systemic inflammation response index (SIRI) are hematological biomarkers reflecting systemic inflammatory burden and have been associated with diabetic complications in patients with T2DM [12,13]. As TNF-α and IL-6 represent molecular components of inflammation, whereas SII and SIRI reflect cellular inflammatory responses, the combined assessment of these markers may provide a more comprehensive understanding of inflammatory status in T2DM. However, prospective studies comparing inflammatory biomarker profiles between metformin monotherapy and metformin plus SGLT-2 inhibitor combination therapy in patients with newly diagnosed T2DM remain limited. Furthermore, comparative evidence from treatment-naïve patients is limited, and potential differences in baseline glycemic status between treatment groups may influence changes in inflammatory biomarkers.

However, prospective studies comparing inflammatory biomarker profiles between metformin monotherapy and metformin plus SGLT-2 inhibitor combination therapy in patients with newly diagnosed T2DM remain limited.

The aim of this prospective observational study was to compare adjusted between-group differences in inflammatory status between patients receiving metformin monotherapy and those receiving metformin plus SGLT-2 inhibitor combination therapy, with TNF-α and IL-6 as the primary inflammatory outcomes and SII and SIRI as secondary inflammatory indices during a six-month follow-up period in patients with newly diagnosed T2DM.

## 2. Materials and Methods

This prospective observational study was conducted at the Internal Medicine Outpatient Clinic of University of Health Sciences, Bağcılar Training and Research Hospital, between January 2026 and June 2026. The primary endpoint was the evaluation of adjusted between-group differences in serum TNF-α and IL-6 levels after six months of metformin monotherapy or metformin plus SGLT-2 inhibitor combination therapy in patients with newly diagnosed T2DM. Secondary endpoints included changes in neutrophil to lymphocyte ratio (NLR), monocyte to lymphocyte ratio (MLR), platelet to lymphocyte ratio (PLR), systemic immune inflammation index (SII), systemic inflammation response index (SIRI), C-reactive protein (CRP), ferritin, triglyceride glucose (TyG) index, uric acid to HDL-cholesterol ratio (UHR), and other metabolic parameters. Analyses of secondary endpoints and correlation analyses were considered exploratory because of the number of comparisons performed, and nominal *p* values were interpreted as hypothesis-generating rather than confirmatory.

The final sample consisted of 64 patients who met the predefined eligibility criteria and completed the six-month follow-up. A total of 64 patients with newly diagnosed T2DM according to the 2025 American Diabetes Association (ADA) diagnostic criteria and without previous antidiabetic treatment were included [14]. Patients with active infection, malignancy, autoimmune or chronic inflammatory disease, chronic liver disease, stage ≥ 3 chronic kidney disease, hematological disease, acute diabetic complications, or medication use that could affect inflammatory responses were excluded.

Thirty-two patients received metformin monotherapy, while 32 patients received metformin plus an SGLT-2 inhibitor combination therapy. The treating physician selected the treatment in routine clinical practice according to individualized glycemic targets and clinical characteristics, in accordance with current ADA recommendations; treatment selection was not randomized. Patients with higher baseline glycated hemoglobin (HbA1c) levels and a greater need for more intensive glycemic management were generally considered for initial combination therapy, whereas patients with lower baseline HbA1c levels were generally treated with metformin monotherapy. Initial combination therapy was considered particularly when baseline HbA1c was substantially above the individualized glycemic target. Metformin was administered at a dose of 1000 mg twice daily, and dapagliflozin or empagliflozin was administered at 10 mg once daily. The study analyzed dapagliflozin and empagliflozin together as an SGLT-2 inhibitor combination group because it was designed to evaluate the class-level association of SGLT-2 inhibitor therapy with inflammatory and metabolic outcomes. Both treatment strategies continued for six months, and clinical and laboratory evaluations were performed at baseline and after six months. Patients with poor treatment adherence, those requiring a change in antidiabetic therapy, or those requiring initiation of additional antidiabetic medication during follow-up were not included in the final analysis. No additional medications, including vitamin D supplementation, were initiated during the six-month follow-up period. Because of the limited sample size, molecule-specific subgroup analyses of dapagliflozin and empagliflozin were not performed.

Blood samples were collected after at least 8 h of fasting. Serum samples were separated by centrifugation at 3000 revolutions per minute (rpm) for 10 min and stored at −80 °C until analysis. Biochemical parameters were measured using a Mindray autoanalyzer (Shenzhen Mindray Bio-Medical Electronics Co., Ltd., Shenzhen, China), hormone parameters were measured using the Siemens ADVIA 1800 autoanalyzer (Siemens Healthineers, Tarrytown, NY, USA), and hematological parameters were assessed using the Sysmex XN-1000 hematology analyzer (Sysmex Corporation, Kobe, Japan). TNF-α was measured using a Human TNF-α enzyme-linked immunosorbent assay (ELISA) Kit (Catalogue No. 201-12-0083), and IL-6 was measured using a Human IL-6 ELISA Kit (Catalogue No. 201-12-0091). Both assays used the double-antibody sandwich ELISA method and followed the manufacturer’s instructions. The assay sensitivity was 2.827 ng/L for TNF-α and 2.112 ng/L for IL-6, with assay ranges of 3–900 ng/L and 3–600 ng/L, respectively. The reported intra-assay and inter-assay coefficients of variation were <10% and <12%, respectively, for both assays. NLR, MLR, PLR, SII [(neutrophil × platelet)/lymphocyte], and SIRI [(neutrophil × monocyte)/lymphocyte] were calculated from complete blood count parameters.

The TyG index was calculated as ln[fasting triglycerides (mg/dL) × fasting plasma glucose (mg/dL)/2]. UHR, triglyceride to HDL-cholesterol ratio (TG/HDL), and estimated glomerular filtration rate (eGFR; Chronic Kidney Disease Epidemiology Collaboration [CKD-EPI] equation) were evaluated.

For the primary inflammatory outcomes, baseline-adjusted multiple linear regression analyses were performed to evaluate between-group differences in six-month TNF-α and IL-6 levels. The same baseline-adjusted approach was applied to CRP, SII, NLR, and selected metabolic parameters.

The Medipol University Faculty of Medicine Non-Interventional Clinical Research Ethics Committee approved the study (Decision No: E-10840098-202.3.02-542; 22.01.2026). The study was conducted in accordance with the principles of the Declaration of Helsinki, and written informed consent was obtained from all participants.

### Statistical Analysis

Statistical analyses were performed using IBM SPSS Statistics for Windows, Version 31.0 (IBM Corp., Armonk, NY, USA). The distributional characteristics of continuous variables were assessed by examining skewness and kurtosis coefficients, and values within the range of ±1.96 were considered compatible with an approximately normal distribution [15]. Continuous variables were presented as mean ± standard deviation and median (minimum–maximum), as appropriate, whereas categorical variables were presented as number (*n*) and percentage (%).

The study compared baseline and six-month measurements between the metformin monotherapy and metformin plus SGLT-2 inhibitor groups using the independent-samples *t*-test for normally distributed variables and the Mann–Whitney U test for non-normally distributed variables. Within-group comparisons between baseline and six-month measurements used the paired-samples *t*-test or Wilcoxon signed-rank test, as appropriate. Changes during follow-up were calculated for biochemical, inflammatory, and metabolic parameters using the formula Δ = 6-month value − baseline value. Spearman rank correlation analysis was used to evaluate associations between treatment group, age, sex, and change scores. Correlation coefficients were interpreted according to their absolute values as low (0.00–0.30), moderate (0.30–0.70), or high (0.70–1.00) correlations.

Because baseline HbA1c levels differed significantly between the treatment groups and the treating physician determined treatment allocation rather than randomization, baseline-adjusted multiple linear regression analyses were performed to account for measured baseline differences. For the primary inflammatory outcomes, TNF-α and IL-6, the 6-month biomarker level served as the dependent variable, and treatment group, age, sex, the corresponding baseline biomarker level, and baseline HbA1c served as independent variables. Thus, the treatment-group coefficient represented the adjusted between-group association in the 6-month biomarker level after accounting for the corresponding baseline biomarker level and the included covariates. The same baseline-adjusted approach was applied to CRP, SII, NLR, and selected metabolic parameters. For the 6-month HbA1c model, treatment group, age, sex, and baseline HbA1c served as independent variables. For the 6-month serum uric acid model, treatment group, age, sex, baseline HbA1c, and baseline serum uric acid served as covariates.

The analyses reported regression coefficients as unstandardized coefficients (B), standard errors (SE), standardized coefficients (β), 95% confidence intervals (95% CIs) for B, and *p* values. Model explanatory performance was assessed using R^2^ and adjusted R^2^, and the F-test evaluated overall model significance. Tolerance and variance inflation factor (VIF) values were used to assess multicollinearity among independent variables; tolerance > 0.10 and VIF < 5 were considered acceptable.

Because TNF-α and IL-6 showed wide distributions and potential influence from extreme observations, sensitivity analyses were performed after natural logarithmic transformation of these biomarkers. The log-transformed 6-month TNF-α and IL-6 levels served as dependent variables in the same baseline-adjusted regression models. These analyses evaluated whether the observed treatment-group associations were robust to the distributional characteristics of the inflammatory biomarkers.

Because vitamin D levels increased significantly in both treatment groups, an additional exploratory analysis assessed whether the change in vitamin D could contribute to the observed cytokine findings. A change score was calculated as Δ vitamin D = 6-month vitamin D − baseline vitamin D, and this variable was additionally entered into the log-transformed TNF-α and IL-6 regression models. These analyses were considered exploratory and were not used to define the primary treatment comparison.

Effect sizes were calculated using Cohen’s d for parametric comparisons and r = |Z|/√N for nonparametric comparisons. For independent-samples *t*-tests, Cohen’s d was calculated using the pooled standard deviation, whereas for paired comparisons it was calculated using the standard deviation of the paired differences. Cohen’s d values of 0.20, 0.50, and 0.80 were interpreted as small, moderate, and large effect sizes respectively. For nonparametric comparisons, r values of 0.10, 0.30, and 0.50 were interpreted as small, moderate, and large associations, respectively.

TNF-α and IL-6 were prespecified as the primary inflammatory outcomes, whereas other biochemical, hematological, and derived inflammatory indices were considered secondary or exploratory outcomes. All statistical tests were two-sided, and a *p* value < 0.05 was considered statistically significant.

## 3. Results

**Figure 1:**
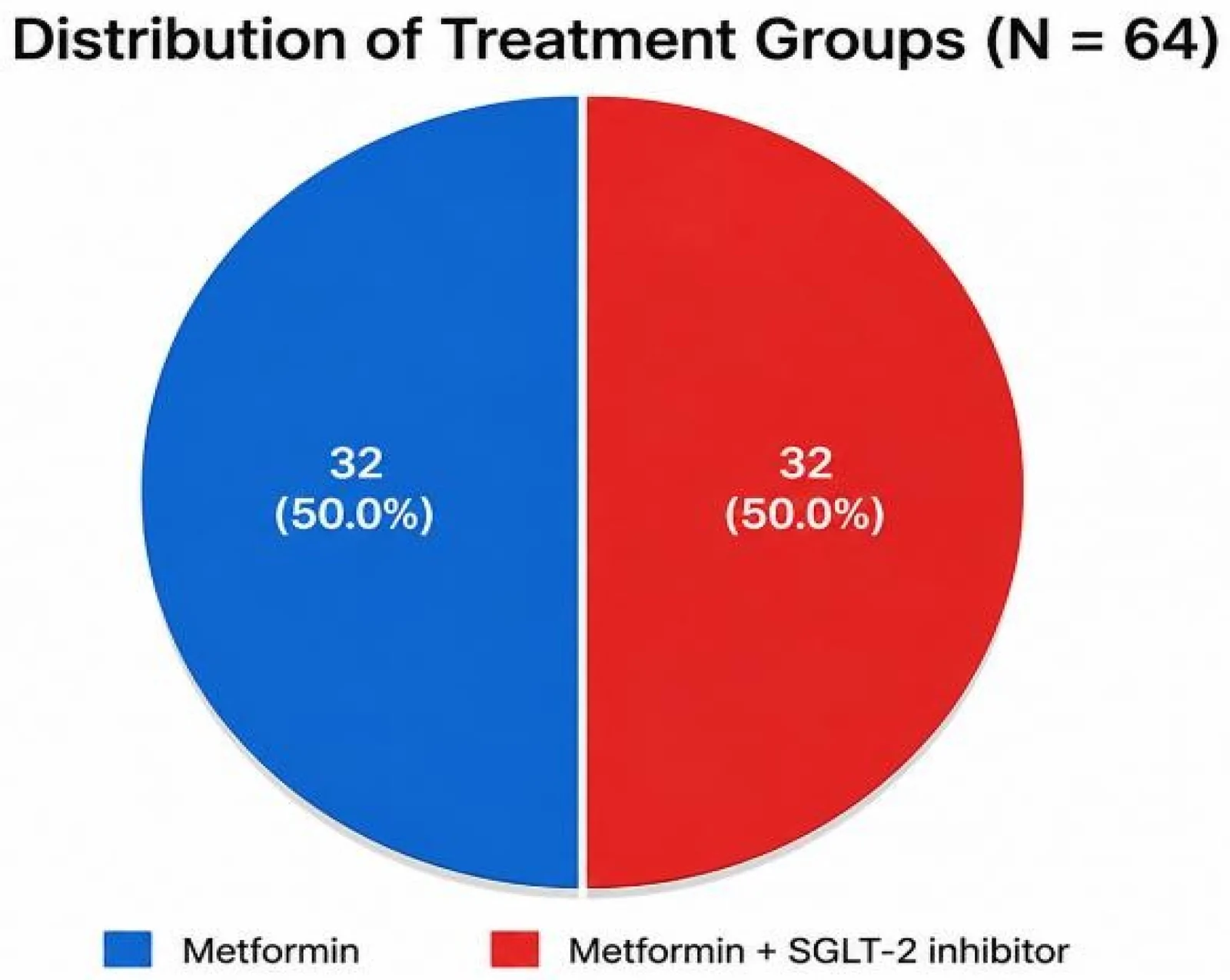
Distribution of treatment groups receiving different SGLT-2 inhibitor therapies.

A total of 64 patients with newly diagnosed T2DM were included in the study; of these 32 patients (50.0%) received metformin monotherapy, while 32 patients (50.0%) received metformin plus an SGLT-2 inhibitor combination therapy (Figure 1). Treatment allocation was physician-directed according to routine clinical practice and individualized glycemic considerations. Consistent with this treatment-selection strategy, baseline HbA1c was significantly higher in the combination therapy group than in the metformin monotherapy group (7.53 ± 0.86% vs. 6.79 ± 0.31%, *p* < 0.001).

Of the 64 patients, 33 (51.6%) were female and 31 (48.4%) were male. No significant differences were observed between the treatment groups in terms of sex distribution or mean age (*p* = 1.000 and *p* = 0.899, respectively). The mean age of the study population was 51.14 ± 8.78 years (Table 1).

At baseline, no significant differences were observed between the treatment groups in TNF-α, IL-6, fasting plasma glucose, CRP, ferritin, or lipid parameters (all *p* > 0.05). However, baseline HbA1c levels were significantly higher in the metformin plus SGLT-2 inhibitor group than in the metformin monotherapy group (7.53 ± 0.86% vs. 6.79 ± 0.31%; *p* < 0.001). At 6-month evaluation, no significant differences were observed between the treatment groups in terms of TNF-α, IL-6, CRP, ferritin, or lipid parameters (all *p* > 0.05). HbA1c levels remained higher in the metformin plus SGLT-2 inhibitor group than in the metformin monotherapy group (6.68 ± 0.73% vs. 6.21 ± 0.45%; *p* = 0.003). However, baseline HbA1c levels were also significantly higher in the combination therapy group (7.53 ± 0.86% vs. 6.79 ± 0.31%; *p* < 0.001). Therefore, the between-group difference in HbA1c at 6 months was interpreted in the context of the baseline imbalance.

Within-group analyses showed significant reductions in TNF-α, IL-6, HbA1c, and fasting plasma glucose levels from baseline to 6 months in both treatment groups (*p* < 0.001). TNF-α levels decreased from 150.29 ± 107.75 to 123.60 ± 118.01 in the metformin group (*p* < 0.001; r = 0.64) and from 134.62 ± 92.63 to 90.07 ± 29.98 in the metformin plus SGLT-2 inhibitor group (*p* < 0.001; r = 0.67). Similarly, IL-6 levels decreased from 108.63 ± 121.16 to 75.78 ± 83.82 in the metformin group (*p* < 0.001; r = 0.72) and from 94.13 ± 91.24 to 48.92 ± 16.71 in the metformin plus SGLT-2 inhibitor group (*p* < 0.001; r = 0.85). These within-group changes do not by themselves indicate that the inflammatory trajectories differed between the treatment groups. HbA1c levels decreased from 6.79 ± 0.31% to 6.21 ± 0.45% in the metformin group and from 7.53 ± 0.86% to 6.68 ± 0.73% in the metformin plus SGLT-2 inhibitor group (both *p* < 0.001), with large within-group effect sizes (Cohen’s d = 1.18 and 1.31, respectively). Fasting plasma glucose levels decreased from 143.08 ± 27.93 to 113.26 ± 21.71 mg/dL in the metformin group (*p* < 0.001; r = 0.70) and from 153.30 ± 39.96 to 127.66 ± 32.09 mg/dL in the metformin plus SGLT-2 inhibitor group (*p* < 0.001; r = 0.62). In contrast, CRP, ferritin, and lipid parameters did not change significantly during the 6-month follow-up period (*p* > 0.05) (Table 2).

According to Table 3, no significant between-group differences were observed in renal function parameters (urea, creatinine, and eGFR) or liver enzymes (lactate dehydrogenase (LDH), aspartate aminotransferase (AST), and alanine aminotransferase (ALT)) at 6 months (all *p* > 0.05). Serum uric acid levels were significantly lower in the metformin plus SGLT-2 inhibitor group than in the metformin group at six months (*p* = 0.016); additionally, uric acid levels increased significantly from baseline in the metformin group (*p* = 0.011). Vitamin D levels increased significantly in both treatment groups at six months (*p* < 0.001). Baseline phosphorus levels were higher in the metformin plus SGLT-2 inhibitor group (*p* = 0.010); however, this difference was no longer significant at six months (*p* = 0.834).

Most hematological parameters did not change significantly during the follow-up period. However, at six months, lymphocyte levels were higher in the metformin plus SGLT-2 inhibitor group than in the metformin group (*p* = 0.037). Hematocrit levels increased from baseline in the metformin plus SGLT-2 inhibitor group compared with baseline (*p* < 0.001). Additionally, sodium levels increased significantly (*p* = 0.008), whereas albumin levels decreased significantly (*p* = 0.006) in the metformin plus SGLT-2 inhibitor group (Table 3).

According to Table 4, the treatment groups did not differ significantly in inflammatory indices (SII, SIRI, PLR, NLR, and MLR) or the metabolic index TyG at baseline or at six months (*p* > 0.05). Within-group analyses also showed no significant changes in any of these parameters during the six-month follow-up period (*p* > 0.05). Regarding UHR, the treatment groups did not differ significantly at baseline or at six months (*p* = 0.075 and *p* = 0.114, respectively). However, UHR levels increased significantly from baseline only in the metformin group (*p* = 0.011), whereas no significant change was observed in the metformin plus SGLT-2 inhibitor group (*p* = 0.066).

**Figure 2:**
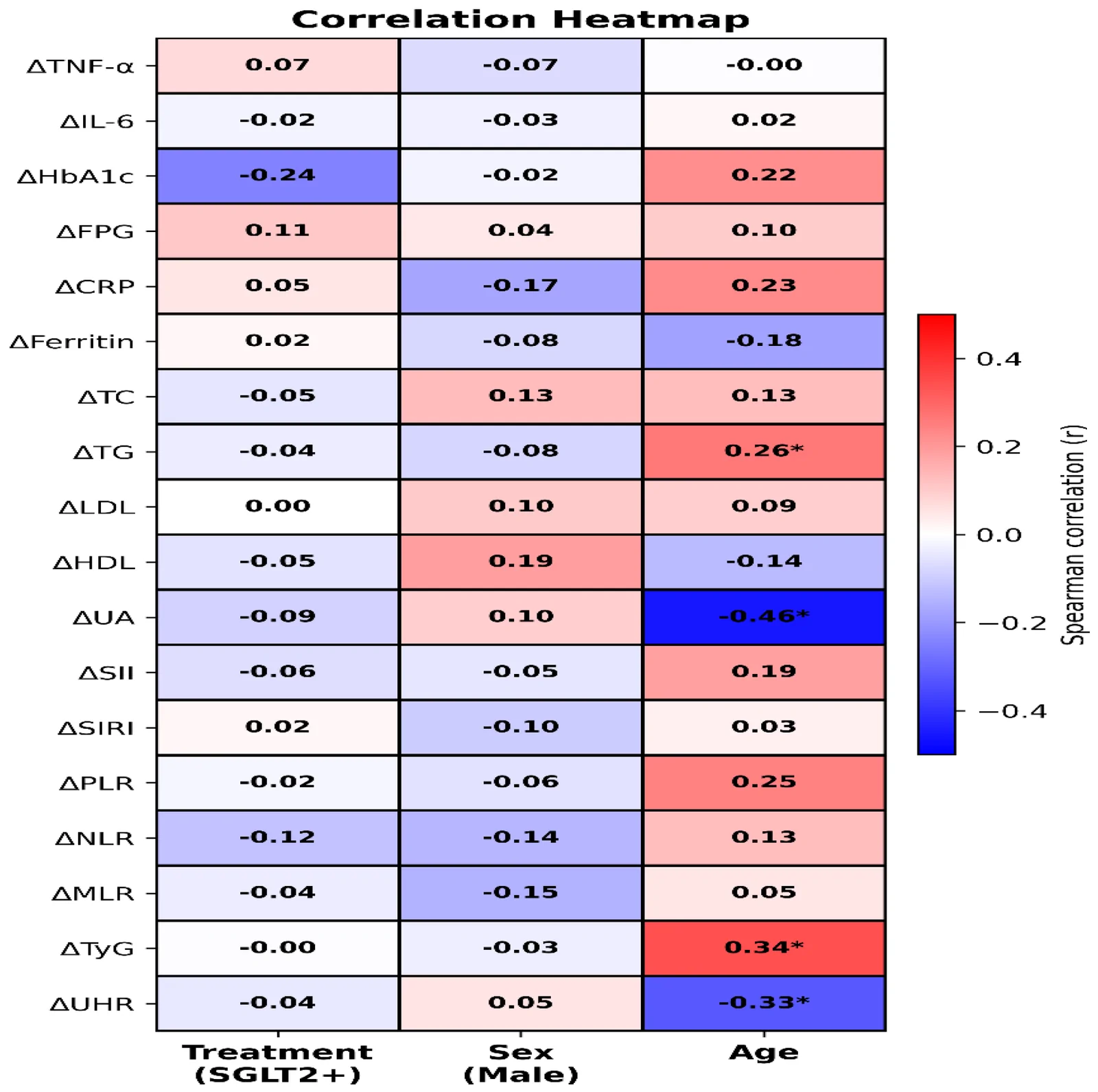
Heatmap of correlations between treatment group, sex, age, and change scores of inflammatory and metabolic parameters. Note: Δ: 6-month value − baseline value. Exploratory Spearman correlation analysis was used to evaluate associations between variables. * *p* < 0.05 was considered statistically significant. r: correlation coefficient; positive values indicate a positive correlation, whereas negative values indicate an inverse correlation. TNF-α: Tumor necrosis factor-alpha; IL-6: interleukin-6; HbA1c: Glycated hemoglobin; FPG: Fasting plasma glucose; CRP: C-reactive protein; TC: Total cholesterol; TG: Triglycerides; LDL: LDL-cholesterol; HDL: HDL-cholesterol; UA: Uric acid; SII: Systemic Immune-Inflammation Index; SIRI: Systemic Inflammation Response Index; PLR: Platelet to Lymphocyte Ratio; NLR: Neutrophil to Lymphocyte Ratio; MLR: Monocyte to Lymphocyte Ratio; TyG: Triglyceride-Glucose Index; UHR: Uric Acid to HDL-Cholesterol Ratio.

Spearman correlation analyses between treatment group, age, sex, and changes in inflammatory and metabolic parameters are presented in Table 5 and Figure 2. The analysis showed no significant associations between treatment group or sex and changes in inflammatory and metabolic parameters (all *p* > 0.05). A nominally borderline inverse association was observed between treatment group and ΔHbA1c (r = −0.244; *p* = 0.056). Age was positively correlated with Δtriglyceride (r = 0.263; *p* = 0.048) and ΔTyG index (r = 0.343; *p* = 0.009), whereas negative correlations were observed between age and Δuric acid (r = −0.457; *p* < 0.001) and ΔUHR (r = −0.326; *p* = 0.013). No significant associations were found between age and the other change scores (all *p* > 0.05).

Multiple linear regression analyses were performed to evaluate adjusted associations between treatment group and 6-month inflammatory and metabolic parameters, accounting for the corresponding baseline parameter, age, sex, and baseline HbA1c. The treatment group was associated with a lower 6-month IL-6 level (B = −37.87; β = −0.31; *p* = 0.038; 95% CI: −73.52 to −2.23), with lower IL-6 levels observed in the metformin plus SGLT-2 inhibitor group than in the metformin monotherapy group. However, the overall IL-6 regression model did not reach statistical significance (F = 2.31; *p* = 0.057); therefore, the statistically significant treatment-group coefficient was interpreted cautiously as a nominal association rather than evidence of a treatment effect. No significant treatment-group associations were observed for the other inflammatory or metabolic parameters (all *p* > 0.05) (Table 6).

Multicollinearity among the independent variables in the multiple linear regression models was assessed using tolerance and variance inflation factor (VIF) values. Tolerance values ranged from 0.56 to 1.00, whereas VIF values ranged from 1.00 to 1.78. All tolerance values were above 0.10, and all VIF values were below 5, indicating no substantial multicollinearity among the independent variables in the regression models.

To assess the potential influence of the wide distributions and extreme values of TNF-α and IL-6 on the observed associations, sensitivity analyses were performed using natural log-transformed baseline and 6-month values of these biomarkers in multiple linear regression models adjusted for age, sex, baseline HbA1c, and the corresponding baseline log-transformed biomarker value (Table 7).

The overall regression models were statistically significant for both log-transformed TNF-α (F = 7.19; *p* < 0.001) and log-transformed IL-6 (F = 6.50; *p* < 0.001). The treatment group was not significantly associated with 6-month log-transformed TNF-α (B = −0.11; β = −0.11; *p* = 0.402), whereas baseline log-transformed TNF-α was positively associated with the 6-month level (B = 0.62; β = 0.61; *p* < 0.001). In contrast, the treatment group was significantly associated with a lower 6-month log-transformed IL-6 level (B = −0.31; β = −0.33; *p* = 0.012), and baseline log-transformed IL-6 was positively associated with the 6-month level (B = 0.34; β = 0.48; *p* < 0.001). After Holm correction for multiple comparisons, the association between treatment group and IL-6 remained statistically significant (adjusted *p* = 0.024), whereas the association for TNF-α remained non-significant (adjusted *p* = 0.402). Given the observational design, non-random treatment allocation, and limited sample size, the IL-6 finding should be interpreted cautiously.

## 4. Discussion

This prospective study included 64 newly diagnosed patients with T2DM who had not previously received any antidiabetic treatment. Patients received either metformin monotherapy (*n* = 32) or metformin plus SGLT-2 inhibitor combination therapy (*n* = 32). After six months of follow-up, significant reductions in TNF-α, IL-6, HbA1c, and fasting plasma glucose levels were observed in both treatment groups. However, no significant unadjusted between-group differences were observed for most inflammatory and metabolic parameters, including TNF-α and IL-6. In the baseline-adjusted multiple linear regression analysis, combination therapy was associated with lower 6-month IL-6 levels, whereas no independent association was observed between treatment group and 6-month TNF-α levels. This association between combination therapy and lower 6-month IL-6 levels persisted in the sensitivity analysis using log-transformed values and remained statistically significant after Holm correction for multiple comparisons. These findings suggest a possible incremental association between SGLT-2 inhibitor-containing therapy and lower IL-6 levels beyond metformin therapy; however, this finding should be interpreted cautiously and cannot be considered evidence of a causal treatment effect. Furthermore, because no established minimum clinically important difference (MCID) has been defined for circulating TNF-α or IL-6 levels in patients with newly diagnosed T2DM, the clinical relevance of the observed differences cannot be determined definitively. The IL-6 finding should therefore be interpreted in the context of the observational design, non-randomized treatment allocation, limited sample size, and the number of secondary and exploratory outcomes evaluated.

The inflammatory associations of metformin have been investigated in different study designs. Meta-analyses have shown that metformin use is particularly associated with reductions in CRP levels; however, its associations with specific cytokines such as TNF-α and IL-6 have been variable [16]. Nevertheless, prospective studies have reported reductions in proinflammatory cytokine levels following metformin treatment. Adeshara et al. reported decreases in TNF-α and IL-6 levels after metformin therapy in patients with type 2 diabetes and suggested that these changes may be related to the anti-inflammatory effects of metformin [17]. Similarly, Mo et al. demonstrated improvements in inflammatory status following metformin treatment in patients with newly diagnosed type 2 diabetes [18]. In the present study, both treatment strategies were associated with reductions in TNF-α and IL-6 levels over six months, consistent with previous reports of reductions in inflammatory markers following metformin treatment. Although the unadjusted between-group comparisons did not demonstrate clear differences, the adjusted analyses suggested a possible additional association between combination therapy and lower IL-6 levels, whereas no corresponding association was observed for TNF-α. These findings suggest that the addition of an SGLT-2 inhibitor may be associated with lower IL-6 levels beyond metformin therapy; however, this interpretation remains tentative because of the observational design and non-randomized treatment allocation.

The associations between SGLT-2 inhibitor therapy and inflammatory markers have primarily been investigated in patients with cardiovascular disease or high cardiometabolic risk. Clinical studies have reported reductions in IL-6 and CRP levels following empagliflozin treatment [19,20]. Furthermore, a meta-analysis found that SGLT-2 inhibitor therapy was associated with lower IL-6 levels, although substantial heterogeneity was observed across the included studies [21].

However, studies directly comparing the addition of an SGLT-2 inhibitor to metformin therapy with metformin monotherapy remain limited. In our study, conducted in newly diagnosed and treatment-naïve T2DM patients, no significant between-group differences were observed in the unadjusted changes in TNF-α or IL-6 levels. In the baseline-adjusted analyses, treatment group was not independently associated with 6-month TNF-α levels, whereas a potential association between combination therapy and lower 6-month IL-6 levels was observed. This association remained significant in the log-transformed sensitivity analysis after Holm correction, whereas no corresponding association was observed for TNF-α. Although experimental studies have reported effects of empagliflozin on NF-κB signaling and proinflammatory cytokine expression [22], these experimental findings cannot be directly extrapolated to changes in circulating inflammatory biomarkers in clinical populations. Moreover, the magnitude and clinical relevance of the anti-inflammatory effects reported in clinical studies remain uncertain because of substantial heterogeneity [21]. Thus, our findings do not provide definitive evidence of an additional anti-inflammatory effect of SGLT-2 inhibitor therapy. However, the baseline-adjusted IL-6 findings suggest a possible treatment-associated reduction that warrants confirmation in larger randomized studies. These findings should be interpreted in the context of the observational design, non-randomized treatment allocation, baseline glycemic imbalance, and relatively short follow-up period. The metabolic and inflammatory effects of SGLT-2 inhibitors may also be considered within the broader liver–kidney metabolic continuum. In T2DM, metabolic dysfunction may involve interconnected hepatic, renal, and systemic inflammatory pathways, while SGLT-2 inhibitors may exert pleiotropic effects beyond glucose lowering, including potential metabolic and renal benefits [23]. These interconnected mechanisms may contribute to the heterogeneous inflammatory responses observed with SGLT-2 inhibitor therapy and warrant further investigation [10].

In our study, significant reductions in HbA1c and fasting plasma glucose levels were observed in both treatment groups. Baseline HbA1c was significantly higher in the combination therapy group than in the metformin monotherapy group, consistent with the physician-directed treatment strategy used in routine clinical practice, whereby patients with a greater need for glycemic control may have been more likely to receive initial combination therapy. Although a greater unadjusted reduction in HbA1c was observed in the combination therapy group, baseline-adjusted analysis did not demonstrate an independent association between treatment group and 6-month HbA1c levels. Therefore, the greater unadjusted reduction observed in the combination therapy group should not be interpreted as evidence of superior glycemic efficacy. Given the observational design and non-randomized treatment allocation, residual confounding cannot be excluded.

Although significant reductions in TNF-α and IL-6 levels were observed in both treatment groups, no significant changes were detected in hemogram-derived inflammatory indices, including SII, SIRI, NLR, PLR, and MLR, and no significant between-group differences were observed in their changes over the six-month follow-up period. These hematological indices should be considered exploratory markers of systemic inflammation rather than direct measures of molecular inflammatory activity. Unlike circulating cytokines such as TNF-α and IL-6, these ratios are nonspecific and reflect the combined contribution of different circulating blood cell populations. Therefore, the absence of significant changes in these indices should not be interpreted as evidence of an absence of changes in inflammatory activity. Rather, circulating cytokines and hemogram-derived inflammatory indices may reflect different dimensions of systemic inflammation. Cytokines represent specific inflammatory mediators that may respond to metabolic interventions, whereas composite hematological indices integrate multiple cellular components and may consequently exhibit different response patterns during short-term treatment. Consistent with previous studies reporting changes in circulating inflammatory cytokines following metformin and SGLT-2 inhibitor therapy [16,17,18,19,20,21], further studies are needed to determine whether changes in hemogram-derived inflammatory indices parallel cytokine responses over longer periods.

In our study, serum uric acid levels decreased significantly in the metformin plus SGLT-2 inhibitor group over the six-month follow-up, whereas an increase from baseline was observed in the metformin group; serum uric acid levels were also lower in the combination therapy group than in the metformin monotherapy group at six months. This finding is consistent with previous evidence showing that SGLT-2 inhibitors reduce serum uric acid levels, possibly through increased renal urate excretion and modulation of renal urate transporters [24,25,26]. However, because dapagliflozin and empagliflozin were pooled in the present study, the observed reduction in serum uric acid cannot be attributed to a specific SGLT-2 inhibitor. Although metformin has also been associated with alterations in uric acid metabolism and lower serum uric acid levels in observational studies [27], no significant reduction was observed in our metformin group. This discrepancy may be related to differences in patient characteristics, treatment duration, or study design. Although changes in serum uric acid may potentially influence the uric acid to HDL-cholesterol ratio (UHR), the clinical interpretation of UHR in the context of antidiabetic treatment remains uncertain because evidence regarding treatment-related changes in UHR is limited.

UHR has been reported as a marker associated with metabolic control and insulin resistance in patients with T2DM [28]; however, evidence regarding changes in UHR following antidiabetic therapy remains limited. In our study, UHR increased significantly only in the metformin monotherapy group after six months of follow-up. However, no significant between-group difference was observed in UHR at six months or in the change from baseline. Therefore, although the reduction in serum uric acid observed with combination therapy may theoretically influence UHR, the present findings do not provide evidence of a significant treatment-related difference in UHR. Further studies are needed to clarify the clinical significance of changes in UHR during antidiabetic treatment.

The observed associations between age and changes in triglycerides, the TyG index, serum uric acid, and UHR suggest that metabolic responses may vary according to individual patient characteristics in addition to treatment strategy. However, given the observational design, these associations should not be interpreted causally. Age-related metabolic alterations and differences in insulin sensitivity may partly contribute to the variability in metabolic responses observed among patients [29].

No significant change in the TyG index was observed after six months of follow-up. The TyG index has been proposed as a practical surrogate marker of insulin resistance and is associated with metabolic status in patients with T2DM [30]. Although metformin and SGLT-2 inhibitors may improve insulin sensitivity and metabolic parameters, these associations may not necessarily translate into significant short-term changes in indirect markers such as the TyG index [31,32]. Because the TyG index is calculated from fasting triglyceride and glucose levels, it may not fully capture short-term changes in insulin sensitivity. In our study, the absence of a significant between-group difference in the change in the TyG index suggests that neither treatment strategy was associated with a clearly different short-term association in this surrogate marker.

A major strength of our study is the prospective evaluation of metformin monotherapy versus metformin plus SGLT-2 inhibitor combination therapy in newly diagnosed, treatment-naïve patients with T2DM selected according to predefined inclusion and exclusion criteria. The study evaluated both molecular inflammatory markers (TNF-α and IL-6) and hematological inflammatory indices (SII and SIRI). In addition, the study directly compared two commonly used treatment strategies and incorporated baseline-adjusted regression analyses to account for baseline differences in HbA1c and other prespecified covariates. Given that previous studies have mainly focused on patients with cardiovascular disease or high cardiometabolic risk, data comparing these two treatment approaches in newly diagnosed T2DM remain limited.

This study has several limitations, including its single-center observational design, small sample size, lack of randomization, and relatively short follow-up period. Because treatment allocation was determined by the treating physician, baseline differences between treatment groups, particularly in HbA1c levels, may have introduced residual confounding despite adjustment for baseline HbA1c and other prespecified covariates. In addition, dapagliflozin and empagliflozin were analyzed as a pooled SGLT-2 inhibitor group. Although this approach allowed evaluation of a class-level association, molecule-specific associations could not be assessed because of the limited sample size. Therefore, the pooled analysis should be considered exploratory and interpreted as a class-level association rather than evidence of a molecule-specific treatment association. Changes in body weight, BMI, lifestyle factors, physical activity, and smoking status during the six-month follow-up were not systematically assessed or recorded as part of the original study design. Therefore, we could not determine whether changes in these factors contributed to the observed changes in inflammatory biomarkers or distinguish direct treatment-related associations from potential indirect associations mediated through weight or other metabolic changes. The relatively small sample size limited the ability to detect modest between-group differences, and small but potentially relevant treatment-associated differences may therefore have remained undetected. In addition, the relatively low R^2^ values observed in several regression models indicate that a substantial proportion of the variability in the outcomes was not explained by the variables included in the models. Therefore, these models should be interpreted primarily as adjusted association models rather than predictive models, and the clinical implications of the observed associations should be interpreted cautiously. Although the association between treatment group and lower 6-month IL-6 levels persisted in the log-transformed sensitivity analysis and remained statistically significant after Holm correction, this finding does not establish causality, and residual confounding cannot be excluded. Finally, given the number of secondary endpoints and correlation analyses, some nominally significant findings may represent chance associations related to multiple testing. Accordingly, these secondary and exploratory findings should be considered hypothesis-generating rather than confirmatory and require validation in larger studies.

In conclusion, both treatment strategies were associated with reductions in TNF-α and IL-6 levels after six months. Although no significant unadjusted between-group differences were observed, baseline-adjusted analyses suggested a potential association between combination therapy and lower 6-month IL-6 levels, while no corresponding association was observed for TNF-α. This IL-6 association persisted in the log-transformed sensitivity analysis and remained statistically significant after Holm correction; however, this finding should not be interpreted causally. Combination therapy was also associated with lower serum uric acid levels at six months. Given the observational design, physician-directed treatment allocation, baseline glycemic imbalance, and limited sample size, these findings should be interpreted as associations and not as evidence of causality. Larger randomized studies are needed to determine whether the addition of an SGLT-2 inhibitor is associated with incremental changes in inflammatory biomarkers beyond metformin therapy.

## Figures and Tables

**Table 1:** Comparison of Demographic Characteristics of Newly Diagnosed Type 2 Diabetes Mellitus Patients According to Treatment Groups.

Variable	Category	Overall (*n* = 64)		Metformin (*n* = 32)		Metformin + SGLT-2 Inhibitor (*n* = 32)		*p*
		*n*	%	*n*	**%**	*n*	%	
Sex	female	33	51.6	16	50.0	17	53.1	1.000
	male	31	48.4	16	50.0	15	46.9	
Age category	<51 years	29	45.3	15	46.9	14	43.8	1.000
	≥51 years	35	54.7	17	53.1	18	56.3	
		**Mean ± SD Med. (Min.–Max.)**		**Mean ± SD** **Med. (Min.–Max.)**		**Mean ± SD** **Med. (Min.–Max.)**		***p***
Age (years)		51.14 ± 8.78 51.00 (32.00–77.00)		51.28 ± 8.28 51.50 (34.00–70.00)		51.00 ± 9.38 51.00 (32.00–77.00)		0.899

Note: Categorical variables were analyzed using the chi-square (χ^2^) test. Continuous variables were analyzed using the independent-samples *t*-test or Mann–Whitney U test, as appropriate according to data distribution.

**Table 2:** Comparison of Inflammatory, Glycemic, and Lipid Parameters According to Treatment Group at Baseline and 6 Months in Newly Diagnosed Patients with Type 2 Diabetes Mellitus.

Variable		Metformin (*n* = 32) Mean ± SD Med (Min–Max)	Metformin + SGLT-2 Inhibitor (*n* = 32) Mean ± SD Med (Min–Max)	*p*_1_
TNF-α (ng/L) (z)	Baseline	150.29 ± 107.75 119.43 (63.67–513.11)	134.62 ± 92.63 118.04 (24.28–598.72)	0.742
	6 months	123.60 ± 118.01 82.87 (45.06–469.80)	90.07 ± 29.98 84.75 (26.76–169.64)	0.666
	*p*_2_	<0.001 *	<0.001 *	
IL-6 (ng/L) (z)	Baseline	108.63 ± 121.16 77.89 (42.87–698.20)	94.13 ± 91.24 74.76 (3.49–484.10)	0.193
	6 months	75.78 ± 83.82 51.19 (30.83–434.10)	48.92 ± 16.71 46.48 (20.41–110.68)	0.077
	*p*_2_	<0.001 *	<0.001 *	
HbA1c (%) (t)	Baseline	6.79 ± 0.31 6.70 (6.30–7.60)	7.53 ± 0.86 7.35 (6.50–9.90)	<0.001 *
	6 months	6.21 ± 0.45 6.20 (4.90–7.00)	6.68 ± 0.73 6.55 (5.60–8.50)	0.003 *
	*p*_2_	<0.001 *	<0.001 *	
Fasting plasma glucose (FPG) (mg/dL) (z)	Baseline	143.08 ± 27.93 134.50 (104.00–250.10)	153.30 ± 39.96 143.40 (101.00–283.00)	0.436
	6 months	113.26 ± 21.71 109.95 (77.00–157.30)	127.66 ± 32.09 120.00 (89.00–228.00)	0.102
	*p*_2_	<0.001 *	<0.001 *	
CRP (mg/L) (z)	Baseline	5.66 ± 7.45 3.50 (2.00–38.10)	8.66 ± 12.54 3.65 (2.00–66.10)	0.444
	6 months	5.92 ± 7.97 3.25 (2.00–37.60)	6.28 ± 7.71 3.90 (2.00–35.00)	0.506
	*p*_2_	0.570	0.895	
Ferritin (µg/L) (t)	Baseline	63.30 ± 48.46 59.05 (6.10–240.50)	80.51 ± 66.29 71.10 (4.90–244.80)	0.243
	6 months	53.20 ± 45.47 38.15 (5.50–178.80)	66.30 ± 47.40 56.90 (11.30–215.10)	0.276
	p_2_	0.121	0.059	
Total cholesterol (mg/dL) (z)	Baseline	214.97 ± 32.36 210.00 (156.00–287.00)	203.82 ± 46.26 201.50 (123.00–360.00)	0.208
	6 months	214.77 ± 45.28 206.00 (137.00–359.00)	211.61 ± 53.86 200.00 (121.00–396.00)	0.525
	*p*_2_	0.530	0.603	
Triglycerides (mg/dL) (z)	Baseline	166.78 ± 91.83 148.50 (46.00–442.00)	188.04 ± 130.42 151.00 (45.00–611.00)	0.830
	6 months	170.87 ± 71.62 157.50 (59.00–349.00)	165.32 ± 72.54 142.00 (14.00–308.00)	0.823
	*p*_2_	0.886	0.741	
LDL-cholesterol (mg/dL) (t)	Baseline	133.28 ± 28.72 130.70 (87.00–184.00)	122.91 ± 34.62 125.80 (44.10–190.00)	0.210
	6 months	133.48 ± 36.79 132.90 (70.60–240.70)	123.47 ± 34.11 126.90 (54.00–181.90)	0.275
	*p*_2_	0.985	0.758	
HDL-cholesterol (mg/dL) (t)	Baseline	50.80 ± 12.55 51.55 (33.80–89.90)	51.57 ± 12.80 49.20 (36.40–82.50)	0.816
	6 months	49.56 ± 11.43 48.85 (32.70–83.40)	49.41 ± 11.87 48.10 (31.60–78.50)	0.962
	*p*_2_	0.683	0.121	

Note: *p*_1_: Between-group comparison at each time point; independent-samples *t*-test or Mann–Whitney U test was used according to data distribution. *p*_2_: Within-group comparison of baseline and 6-month values; paired-samples *t*-test or Wilcoxon signed-rank test was used according to data distribution. * *p* < 0.05 indicates statistical significance.

**Table 3:** Comparison of Renal, Hepatic, Hematological, and Electrolyte Parameters at Baseline and 6 Months in Patients with Newly Diagnosed Type 2 Diabetes Mellitus.

Variable		Metformin (*n* = 32) Mean ± SD Med (Min–Max)	Metformin + SGLT-2 Inhibitor (*n* = 32) Mean ± SD Med (Min–Max)	*p*_1_
Urea (mg/dL) (t)	Baseline	31.41 ± 7.37 30.00 (18.00–46.00)	31.94 ± 8.01 31.50 (19.00–54.00)	0.783
	6 months	32.63 ± 6.66 32.50 (18.00–44.00)	33.72 ± 7.43 34.00 (18.00–48.00)	0.548
	*p*_2_	0.667	0.194	
Creatinine (mg/dL) (t)	Baseline	0.88 ± 0.12 0.85 (0.70–1.14)	0.88 ± 0.13 0.85 (0.62–1.13)	0.954
	6 months	0.89 ± 0.17 0.87 (0.61–1.27)	0.84 ± 0.15 0.80 (0.63–1.20)	0.259
	*p*_2_	0.708	0.152	
Estimated glomerular filtration rate (eGFR) (mL/min/1.73 m^2^) (t)	Baseline	88.47 ± 12.48 88.50 (65.00–115.00)	86.00 ± 14.63 84.00 (63.00–114.00)	0.471
	6 months	86.93 ± 16.08 87.00 (57.00–114.00)	89.39 ± 13.89 88.50 (62.00–115.00)	0.521
	*p*_2_	0.680	0.127	
Uric acid (mg/dL) (t)	Baseline	5.30 ± 1.38 5.15 (2.50–8.10)	4.70 ± 1.12 4.75 (1.70–7.70)	0.060
	6 months	5.82 ± 1.38 5.40 (3.30–8.50)	5.04 ± 1.08 4.95 (2.70–7.50)	0.016 *
	*p*_2_	0.011 *	0.135	
LDH (U/L) (t) (Lactate dehydrogenase)	Baseline	188.06 ± 25.78 193.50 (130.00–242.00)	190.59 ± 36.48 190.00 (127.00–265.00)	0.750
	6 months	183.90 ± 26.73 179.00 (138.00–239.00)	190.84 ± 33.30 183.00 (146.00–295.00)	0.371
	*p*_2_	0.587	0.971	
AST (U/L) (z) (Aspartate aminotransferase)	Baseline	22.53 ± 8.15 19.50 (14.00–53.00)	20.94 ± 7.35 19.00 (12.00–41.00)	0.404
	6 months	22.83 ± 13.32 21.00 (12.00–87.00)	21.25 ± 5.35 20.00 (13.00–38.00)	0.938
	*p*_2_	0.579	0.247	
ALT (U/L) (z) (Alanine aminotransferase)	Baseline	26.28 ± 16.15 21.50 (12.00–83.00)	25.75 ± 11.88 22.50 (11.00–60.00)	0.672
	6 months	26.77 ± 17.63 22.00 (9.00–81.00)	27.00 ± 11.09 27.00 (10.00–54.00)	0.317
	*p*_2_	0.797	0.351	
Vitamin D (ng/mL) (z)	Baseline	11.96 ± 6.74 10.50 (4.20–26.40)	9.17 ± 5.90 7.65 (4.20–35.50)	0.175
	6 months	22.34 ± 9.54 19.50 (7.20–43.10)	20.02 ± 11.23 19.85 (4.20–48.50)	0.321
	*p*_2_	<0.001 *	<0.001 *	
Calcium (mg/dL) (t)	Baseline	9.81 ± 0.36 9.75 (9.00–10.50)	9.75 ± 0.49 9.70 (8.90–10.80)	0.580
	6 months	9.93 ± 0.34 9.95 (9.00–10.60)	9.96 ± 0.45 10.00 (8.80–10.80)	0.762
	*p*_2_	0.202	0.065	
Phosphorus (mg/dL) (t)	Baseline	3.27 ± 0.54 3.40 (2.00–4.40)	3.68 ± 0.69 3.65 (2.40–5.20)	0.010 *
	6 months	3.56 ± 0.54 3.60 (2.40–4.60)	3.58 ± 0.48 3.55 (2.70–4.90)	0.834
	*p*_2_	0.028 *	0.486	
Magnesium (mg/dL) (t)	Baseline	2.09 ± 0.15 2.13 (1.74–2.31)	2.11 ± 0.18 2.11 (1.77–2.47)	0.585
	6 months	2.13 ± 0.15 2.15 (1.83–2.35)	2.15 ± 0.18 2.18 (1.82–2.56)	0.654
	*p*_2_	0.191	0.419	
Sodium (mmol/L) (t)	Baseline	140.33 ± 2.48 140.45 (134.90–145.00)	140.07 ± 2.55 140.10 (135.00–146.10)	0.678
	6 months	140.73 ± 2.14 140.35 (137.00–146.20)	141.24 ± 2.65 141.80 (134.60–146.50)	0.409
	*p*_2_	0.380	0.008 *	
Potassium (mmol/L) (t)	Baseline	4.54 ± 0.31 4.54 (3.93–5.24)	4.51 ± 0.35 4.55 (3.72–5.18)	0.655
	6 months	4.57 ± 0.40 4.52 (4.00–5.59)	4.49 ± 0.43 4.47 (3.30–5.40)	0.468
	*p*_2_	0.699	0.843	
White blood cell count (WBC) (×10^3^/µL) (t)	Baseline	7.62 ± 1.69 7.31 (4.68–11.10)	8.13 ± 1.47 7.98 (4.96–11.00)	0.212
	6 months	7.46 ± 1.61 7.14 (5.28–10.85)	7.92 ± 1.39 7.85 (5.36–10.16)	0.229
	*p*_2_	0.440	0.452	
Neutrophil count (×10^3^/µL) (t)	Baseline	4.39 ± 1.29 4.04 (2.65–7.35)	4.58 ± 0.96 4.44 (2.00–6.81)	0.504
	6 months	4.35 ± 1.24 4.02 (2.90–7.91)	4.44 ± 1.07 4.59 (2.31–6.64)	0.760
	*p*_2_	0.775	0.474	
Monocyte count (×10^3^/µL) (z)	Baseline	0.46 ± 0.11 0.45 (0.22–0.67)	0.46 ± 0.16 0.43 (0.19–0.78)	0.673
	6 months	0.43 ± 0.11 0.44 (0.22–0.72)	0.45 ± 0.16 0.42 (0.21–1.02)	0.971
	*p*_2_	0.088	0.217	
Monocyte percentage (%) (z)	Baseline	6.15 ± 1.39 6.10 (2.80–9.40)	5.71 ± 1.94 5.55 (2.90–12.00)	0.212
	6 months	5.85 ± 1.30 5.90 (3.90–8.50)	5.66 ± 1.68 5.55 (3.60–11.70)	0.329
	*p*_2_	0.336	0.327	
Lymphocyte count (×10^3^/µL) (t)	Baseline	2.50 ± 0.53 2.48 (1.49–3.58)	2.85 ± 0.91 2.95 (0.95–4.60)	0.073
	6 months	2.44 ± 0.55 2.39 (1.23–3.56)	2.78 ± 0.67 2.71 (1.72–4.19)	0.037 *
	*p*_2_	0.500	0.553	
Hematocrit (%) (t)	Baseline	41.16 ± 4.05 41.30 (34.30–50.10)	42.37 ± 4.07 42.85 (35.40–50.60)	0.246
	6 months	42.38 ± 3.83 42.20 (35.50–50.10)	43.99 ± 4.78 43.60 (36.00–53.50)	0.154
	*p*_2_	0.056	<0.001 *	
Plateletcrit (%) (t)	Baseline	0.26 ± 0.06 0.25 (0.14–0.42)	0.27 ± 0.06 0.26 (0.16–0.40)	0.640
	6 months	0.25 ± 0.05 0.24 (0.16–0.37)	0.27 ± 0.06 0.28 (0.17–0.40)	0.255
	*p*_2_	0.233	0.589	
Platelet count (×10^3^/µL) (t)	Baseline	264.17 ± 51.26 254.50 (177.00–395.00)	270.34 ± 67.60 268.00 (157.00–433.00)	0.688
	6 months	261.10 ± 52.37 248.00 (169.00–395.00)	275.00 ± 61.13 277.50 (167.00–433.00)	0.347
	*p*_2_	0.490	0.462	
TSH (mIU/L) (t) (Thyroid-stimulating hormone)	Baseline	2.09 ± 1.16 1.89 (0.02–4.17)	1.90 ± 1.01 1.69 (0.49–4.00)	0.476
	6 months	2.03 ± 1.29 1.84 (0.24–5.10)	1.67 ± 0.91 1.55 (0.20–4.85)	0.218
	*p*_2_	0.585	0.277	
Albumin (g/dL) (t)	Baseline	4.68 ± 0.22 4.67 (4.15–5.14)	4.71 ± 0.27 4.70 (4.32–5.44)	0.678
	6 months	4.60 ± 0.29 4.58 (4.00–5.06)	4.57 ± 0.28 4.60 (4.00–5.26)	0.689
	*p*_2_	0.123	0.006 *	

Note: *p*_1_ indicates the comparison between treatment groups at each time point. For normally distributed variables, the independent samples *t*-test was used, whereas the Mann–Whitney U test was used for non-normally distributed variables. *p*_2_ indicates the within-group comparison between baseline and 6-month measurements. For normally distributed variables, the paired samples *t*-test was applied, whereas for non-normally distributed variables, the Wilcoxon signed-rank test was used. The symbols (t) and (z) indicate the statistical test used for between-group comparisons: (t) denotes the independent samples *t*-test, and (z) denotes the Mann–Whitney U test. * *p* < 0.05 indicates statistical significance.

**Table 4:** Comparison of Inflammatory and Metabolic Indices Between Treatment Groups at Baseline and 6 Months in Newly Diagnosed Patients with Type 2 Diabetes Mellitus.

Variable		Metformin (*n* = 32) Mean ± SD Median (Min.–Maks.)	Metformin + SGLT-2 Inhibitor (*n* = 32) Mean ± SD Median (Min.–Maks.)	*p*_1_
SII (z)	Baseline	467.41 ± 141.30 484.89 (243.24–799.74)	476.30 ± 209.14 446.28 (138.33–1119.74)	0.735
	6 months	474.95 ± 165.20	457.78 ± 169.37	0.554
		447.72 (266.54–1101.99)	409.65 (181.11–773.16)	
	*p*_2_	0.855	0.627	
SIRI (z)	Baseline	0.825 ± 0.33	0.805 ± 0.52	0.602
		0.746 (0.393–1.676)	0.721 (0.303–3.374)	
	6 months	0.791 ± 0.32	0.771 ± 0.53	0.305
		0.689 (0.362–1.668)	0.657 (0.292–3.345)	
	*p*_2_	0.554	0.350	
PLR (t)	Baseline	108.65 ± 25.22 108.09 (70.92–170.16)	102.85 ± 35.52 93.96 (57.48–203.96)	0.464
	6 months	112.44 ± 33.59	102.73 ± 28.41	0.227
		97.46 (61.80–184.11)	101.89 (62.77–191.59)	
	*p*_2_	0.387	0.985	
NLR (z)	Baseline	1.781 ± 0.49	1.835 ± 0.98	0.375
		1.711 (1.064–2.915)	1.493 (0.881–5.436)	
	6 months	1.837 ± 0.53	1.670 ± 0.53	0.189
		1.790 (1.006–3.380)	1.508 (0.897–3.279)	
	*p*_2_	0.785	0.501	
MLR (z)	Baseline	0.188 ± 0.05 0.177 (0.099–0.295)	0.178 ± 0.11 0.156 (0.080–0.792)	0.065
	6 months	0.181 ± 0.04	0.169 ± 0.08	0.059
		0.176 (0.114–0.325)	0.146 (0.089–0.593)	
	*p*_2_	0.530	0.603	
TyG Index (t)	Baseline	9.222 ± 0.64	9.363 ± 0.68	0.418
		9.250 (7.780–10.375)	9.225 (8.400–10.898)	
	6 months	9.076 ± 0.51	9.113 ± 0.69	0.814
		9.114 (7.906–10.212)	9.200 (6.498–10.121)	
	*p*_2_	0.083	0.087	
UHR (t)	Baseline	0.112 ± 0.04 0.114 (0.036–0.208)	0.095 ± 0.03 0.095 (0.044–0.151)	0.075
	6 months	0.124 ± 0.04	0.109 ± 0.034	0.114
		0.122 (0.043–0.223)	0.100 (0.060–0.200)	
	*p*_2_	0.011 *	0.066	

Note: *p*_1_: Comparison of measurements between treatment groups at each time point; the independent-samples *t*-test was used for normally distributed variables, and the Mann–Whitney U test was used for non-normally distributed variables. *p*_2_: Within-group comparison of baseline and 6-month measurements; the paired-samples *t*-test was used for normally distributed variables, and the Wilcoxon signed-rank test was used for non-normally distributed variables. The symbols (t) and (z) indicate the statistical test used for between-group comparisons: (t) denotes the independent-samples *t*-test, whereas (z) denotes the Mann–Whitney U test. * *p* < 0.05 indicates statistical significance. SII: systemic immune-inflammation index; SIRI: systemic inflammation response index; PLR: platelet-to-lymphocyte ratio; NLR: neutrophil-to-lymphocyte ratio; MLR: monocyte-to-lymphocyte ratio; TyG: triglyceride-glucose index; UHR: uric acid-to-HDL-cholesterol ratio.

**Table 5:** Correlation Analysis Results Between Treatment Group, Sex, Age, and Change Scores of Inflammatory and Metabolic Parameters.

Variable	Treatment Group	Membership in the Metformin + SGLT-2 Inhibitor Group	Male	Age (Year)
ΔTNF-α	r	0.072	−0.070	−0.004
	*p*	0.590	0.600	0.973
ΔIL-6	r	−0.020	−0.028	0.017
	*p*	0.881	0.834	0.898
ΔHbA1c	r	−0.244	−0.023	0.220
	*p*	0.056	0.862	0.085
ΔFasting plasma glucose (FPG)	r	0.109	0.043	0.100
	*p*	0.399	0.738	0.439
ΔCRP	r	0.049	−0.175	0.228
	*p*	0.710	0.178	0.077
ΔFerritin	r	0.019	−0.076	−0.180
	*p*	0.884	0.563	0.170
ΔTotal cholesterol	r	−0.048	0.130	0.127
	*p*	0.723	0.334	0.348
ΔTriglyceride	r	−0.038	−0.080	0.263 *
	*p*	0.776	0.554	0.048
ΔLDL-cholesterol	r	0.000	0.103	0.094
	*p*	1.000	0.448	0.487
ΔHDL-cholesterol	r	−0.051	0.190	−0.136
	*p*	0.705	0.157	0.314
ΔUric acid	r	−0.088	0.097	−0.457 *
	*p*	0.494	0.455	<0.001
ΔSII	r	−0.064	−0.048	0.186
	*p*	0.629	0.714	0.155
ΔSIRI	r	0.017	−0.100	0.029
	*p*	0.895	0.446	0.825
ΔPLR	r	−0.019	−0.058	0.246
	*p*	0.884	0.661	0.058
ΔNLR	r	−0.118	−0.143	0.127
	*p*	0.371	0.277	0.335
ΔMLR	r	−0.039	−0.150	0.047
	*p*	0.770	0.251	0.719
ΔTyG Index	r	−0.004	−0.032	0.343 *
	*p*	0.975	0.813	0.009
ΔUHR	r	−0.043	0.053	−0.326 *
	*p*	0.752	0.693	0.013

Note: Δ: Change score calculated as the difference between the 6-month and baseline measurements (Δ = 6 months − baseline). r: Spearman correlation coefficient. Exploratory Spearman correlation analyses were performed to evaluate associations between variables. * A *p*-value < 0.05 was considered statistically significant. The correlation coefficient (r) indicates the direction and strength of the association. Treatment group was coded as a binary variable (metformin = 0, metformin + SGLT-2 inhibitor = 1), and sex was coded as a binary variable (female = 0, male = 1). SII: Systemic Immune-Inflammation Index; SIRI: Systemic Inflammation Response Index; PLR: Platelet to Lymphocyte Ratio; NLR: Neutrophil to Lymphocyte Ratio; MLR: Monocyte to Lymphocyte Ratio; TyG: Triglyceride-Glucose Index; UHR: Uric Acid to HDL-Cholesterol Ratio.

**Table 6:** Multivariable Linear Regression Analysis of 6-Month Inflammatory and Metabolic Outcomes.

Dependent Variable	Independent Variable	B	SE	β	*p*	95% CI for B	R^2^	Adjusted R^2^	Model F; *p*
TNF-α (6 months)	Treatment group	−26.80	21.23	−0.15	0.213	−69.39; 15.80	0.42	0.36	7.64 <0.001
	Age	0.75	1.01	0.08	0.461	−1.28; 2.78			
	Sex	4.35	18.38	0.03	0.814	−32.53; 41.22			
	Baseline HbA1c	4.57	14.06	0.04	0.747	−23.63; 32.77			
	Baseline TNF-α	0.51	0.09	0.61	<0.001 *	0.33; 0.69			
IL-6 (6 months)	Treatment group	−37.87	17.77	−0.31	0.038 *	−73.52; −2.23	0.18	0.10	2.31 0.057
	Age	0.60	0.85	0.09	0.487	−1.11; 2.30			
	Sex	−6.08	15.39	−0.05	0.694	−36.95; 24.79			
	Baseline HbA1c	17.63	11.82	0.22	0.142	−6.07; 41.33			
	Baseline IL-6	0.16	0.07	0.28	0.031 *	0.02; 0.30			
CRP (6 months)	Treatment group	0.06	2.14	0.00	0.977	−4.23; 4.35	0.22	0.15	3.12 0.015
	Age	−0.08	0.11	−0.10	0.438	−0.29; 0.13			
	Sex	0.12	1.85	0.01	0.949	−3.59; 3.83			
	Baseline HbA1c	−0.92	1.44	−0.09	0.529	−3.81; 1.98			
	Baseline CRP	0.33	0.09	0.45	<0.001 *	0.15; 0.52			
SII (6 months)	Treatment group	−36.94	47.56	−0.11	0.441	−132.28; 58.41	0.18	0.10	2.34 0.054
	Age	−0.19	2.43	−0.01	0.939	−5.06; 4.69			
	Sex	−51.94	42.02	−0.16	0.222	−136.19; 32.30			
	Baseline HbA1c	22.16	31.66	0.10	0.487	−41.32; 85.64			
	Baseline SII	0.33	0.12	0.36	0.008 *	0.09; 0.58			
NLR (6 months)	Treatment group	−0.20	0.15	−0.18	0.207	−0.50; 0.11	0.19	0.12	2.53 0.039
	Age	−0.004	0.008	−0.06	0.636	−0.019; 0.012			
	Sex	−0.13	0.13	−0.12	0.327	−0.40; 0.14			
	Baseline HbA1c	0.03	0.10	0.04	0.802	−0.18; 0.23			
	Baseline NLR	0.26	0.09	0.37	0.004 *	0.08; 0.43			
HbA1c (6 months)	Treatment group	0.06	0.15	0.05	0.701	−0.24; 0.36	0.44	0.40	11.22 <0.001
	Age	0.007	0.007	0.10	0.328	−0.007; 0.022			
	Sex	−0.05	0.13	−0.04	0.684	−0.31; 0.21			
	Baseline HbA1c	0.55	0.10	0.64	<0.001 *	0.35; 0.75			
Fasting glucose (6 months)	Treatment group	−2.12	7.28	−0.04	0.773	−16.70; 12.47	0.31	0.25	5.00 <0.001
	Age	0.14	0.36	0.04	0.696	−0.57; 0.85			
	Sex	−5.59	6.34	−0.10	0.382	−18.29; 7.11			
	Baseline HbA1c	21.94	5.23	0.58	<0.001 *	11.46; 32.41			
	Baseline fasting glucose	−0.02	0.10	−0.03	0.835	−0.22; 0.18			
TyG index (6 months)	Treatment group	−0.13	0.17	−0.11	0.442	−0.48; 0.21	0.25	0.18	3.46 0.009
	Age	0.013	0.008	0.20	0.114	−0.003; 0.030			
	Sex	0.05	0.15	0.04	0.759	−0.25; 0.34			
	Baseline HbA1c	0.14	0.12	0.18	0.229	−0.09; 0.38			
	Baseline TyG	0.41	0.12	0.45	<0.001 *	0.18; 0.65			
Uric acid (6 months)	Treatment group	−0.51	0.27	−0.20	0.065	−1.04; 0.03	0.56	0.52	14.32 <0.001
	Age	−0.05	0.01	−0.35	<0.001 *	−0.08; −0.03			
	Sex	0.41	0.23	0.16	0.089	−0.06; 0.88			
	Baseline HbA1c	0.15	0.18	0.09	0.398	−0.21; 0.51			
	Baseline uric acid	0.62	0.10	0.60	<0.001 *	0.42; 0.82			
UHR (6 months)	Treatment group	−0.003	0.008	−0.04	0.740	−0.019; 0.014	0.60	0.56	15.06 <0.001
	Age	−0.001	0.000	−0.26	0.005 *	−0.002; 0.000			
	Sex	0.014	0.007	0.19	0.062	−0.001; 0.029			
	Baseline HbA1c	0.001	0.005	0.01	0.903	−0.010; 0.011			
	Baseline UHR	0.620	0.103	0.60	<0.001 *	0.413; 0.827			
HDL-cholesterol (6 months)	Treatment group	−2.85	2.38	−0.13	0.235	−7.62; 1.92	0.58	0.54	14.11 <0.001
	Age	−0.04	0.12	−0.03	0.762	−0.27; 0.20			
	Sex	−0.62	2.19	−0.03	0.779	−5.00; 3.77			
	Baseline HbA1c	0.97	1.61	0.07	0.549	−2.26; 4.19			
	Baseline HDL	0.67	0.09	0.77	<0.001 *	0.49; 0.85			
Triglycerides (6 months)	Treatment group	−5.45	21.11	−0.04	0.797	−47.83; 36.93	0.23	0.15	3.03 0.018
	Age	0.60	1.02	0.07	0.558	−1.45; 2.66			
	Sex	5.65	18.31	0.04	0.759	−31.11; 42.40			
	Baseline HbA1c	−5.36	14.23	−0.06	0.708	−33.92; 23.20			
	Baseline triglycerides	0.32	0.08	0.48	<0.001 *	0.15; 0.48			

Note: B, unstandardized regression coefficient; SE, standard error; β, standardized regression coefficient; CI, confidence interval; R^2^, coefficient of determination; SII: systemic immune-inflammation index; NLR: neutrophil-to-lymphocyte ratio; TyG: triglyceride-glucose index; UHR: uric acid to HDL-cholesterol ratio; CRP: C-reactive protein; TNF-α: tumor necrosis factor-alpha; IL-6: interleukin-6. * A *p*-value < 0.05 was considered statistically significant.

**Table 7:** Sensitivity analysis of the association between treatment group and 6-Month TNF-α and IL-6 levels using log-transformed multiple linear regression models adjusted for baseline values.

Dependent Variable	Independent Variable	B	SE	β	*p*	95% C for B	R^2^	Adjusted R^2^	Model F *p*
TNF-α (6 months)	Treatment group	−0.11	0.13	−0.11	0.402	−0.36; 0.15	0.40	0.35	7.19 <0.001 *
	Age	0.001	0.006	0.02	0.864	−0.011; 0.013			
	Sex	0.005	0.108	0.01	0.960	−0.212; 0.223			
	Baseline HbA1c	0.07	0.08	0.11	0.386	−0.09; 0.24			
	Baseline TNF-α	0.62	0.11	0.61	<0.001 *	0.40; 0.84			
IL-6 (6 months)	Treatment group	−0.31	0.12	−0.33	0.012 *	−0.55; −0.07	0.38	0.32	6.50 <0.001 *
	Age	0.004	0.006	0.08	0.448	−0.007; 0.016			
	Sex	−0.012	0.102	−0.01	0.908	−0.216; 0.192			
	Baseline HbA1c	0.15	0.08	0.24	0.069	−0.01; 0.31			
	Baseline IL-6	0.34	0.08	0.48	<0.001 *	0.18; 0.50			

Note: B, unstandardized regression coefficient; SE, standard error; β, standardized regression coefficient; CI, confidence interval. Treatment group was coded as metformin = 0 and metformin + SGLT-2 inhibitor = 1; sex was coded as female = 0 and male = 1. Constant terms are not shown. * A *p*-value < 0.05 was considered statistically significant. TNF-α: tumor necrosis factor-alpha; IL-6: interleukin-6.

## Data Availability

Data is unavailable due to privacy or ethical restrictions.
